# Tracking Acquisition of Language in Kids (TALK) study protocol: A longitudinal investigation of infants at high vs. low risk for atypical speech and language development

**DOI:** 10.1371/journal.pone.0335596

**Published:** 2026-01-28

**Authors:** Gina Goble, Victoria Hennessy, Tzu-Han Zoe Cheng, Amy Rodda, Samu Taulu, Reyna L. Gordon, T. Christina Zhao

**Affiliations:** 1 Institute for Learning and Brain Sciences, University of Washington, Seattle, Washington, United States of America; 2 Department of Cognitive Science, UC San Diego, San Diego, California, United States of America; 3 Department of Speech and Hearing Sciences, University of Washington, Seattle, Washington, United States of America; 4 Department of Physics, University of Washington, Seattle, Washington, United States of America; 5 Department of Otolaryngology, Vanderbilt University Medical Center, Nashville, Tennessee, United States of America; 6 Department of Hearing & Speech Sciences, Vanderbilt University Medical Center, Nashville, Tennessee, United States of America; Public Library of Science, UNITED STATES OF AMERICA

## Abstract

The *sensitive period* for phonetic learning, normally considered to be between 6–12 months of age, has been demonstrated as one of the earliest milestones for language development. Infant speech processing towards the end of the *sensitive period* has been shown to predict individual language development trajectories up to school entry and most recently, risk of speech and language disorders, suggesting its potential clinical relevance. Yet, this literature is largely limited to typically developing infants with regard to their family histories of speech and language delays or disorders. The current study begins to fill the gap by investigating associations between neural markers of the *sensitive period*, family history risk factors, and language outcomes by gathering extensive information from a large group of infants. Specifically, family information includes an extensive parental survey of family background and clinical history, a comprehensive assessment of language skills for one older sibling by a research speech-language pathologist (SLP), and a daylong audio recording in infants’ homes for assessing their language environment. The study design focuses on comparing neural predictors of language development in infants with or without first-degree family history of speech and language delays and disorders (i.e., High vs. Low-Risk infants). Infants’ neural speech processing is measured three times using Magnetoencephalography at 6 months, 12 months, and 14 months of age. Infants’ language development is tracked until school entry by both parental surveys and the same comprehensive assessment protocol with a research SLP. This protocol documents the study design and methodological details for data collection and preprocessing. This study will allow our research team to start tackling important questions regarding early predictors of speech and language delays and disorders (e.g., late-talking, Developmental Language Disorder) and contribute significant value to the broader field.

## Introduction

The *sensitive period* for phonetic learning, roughly between 6–12 months of age, is well established as one of the earliest milestones in typical language development [1]. Converging evidence suggests that the learning outcomes from this period are reliable predictors of individual language skills later in life, such as vocabulary size and grammar skills [1–3]. Most recently, a small preliminary study extended this research and demonstrated that infants’ neural processing of speech towards the end of the *sensitive period* could predict not only individual expressive grammar skills at 6 years of age, but also the risk of developing speech and language disorders [4]. These initial findings suggest that the functional speech processing during the *sensitive period* may have important clinical relevance, with the potential to mark atypical language development and detect risk for later developmental speech and language disorders. However, this line of existing research has largely focused on typically developing infants without taking their family history of language disorders into account. At this juncture, there is a critical need to better understand the *sensitive period*, particularly its underlying neural mechanisms, in infants who are at an elevated risk of developing language-related disorders (i.e., High-Risk infants) such as developmental language disorder (DLD) (i.e., infants with first-degree family history of speech and language delay and disorders) in comparison to Low-Risk infants.

### Atypical speech and language development

Speech and language delay and disorders can affect 3–16 percent of children [5]. While some have a known cause, such as hearing loss (13.3%), many do not have an identifiable origin [6]. Developmental Language Disorder (DLD) represents a prominent example as it is a highly prevalent condition where individuals can exhibit deficits in receptive or expressive language without known cause. It has previously been described by many terms, such as ‘Specific Language Impairment (SLI)’ and ‘Primary Language Impairment’. Most recently, there was an effort by the CATALISE consortium [7–9] to establish consensus among experts of different backgrounds, leading to the current label ‘Developmental Language Disorder (DLD)’. It was previously estimated that SLI can affect at least 7% of the population [10]. Affected children can experience long-term problems, including behavioral, psychiatric, emotional, academic and social adaptation [9,11–13]. Children may begin to show language deficits as early as infancy, but DLD cannot be reliably diagnosed until age four [14], meaning the critical period in which intervention could be the most effective may be missed. Additionally, the majority of DLD cases in children are thought to remain unidentified, further indicating a great need to improve the early detection process by establishing risk factors [10,15–17].

DLD has been shown to have a genetic component such that individuals have a significantly elevated risk of developing DLD if there is a familial history [18–20]. For example, close to half of the siblings of individuals with DLD (SLI) exhibit difficulties in language [21], and infant siblings of children with DLD (SLI) scored significantly lower on language measures when they were tested at 3 years of age, compared to infant siblings of children without SLI [22]. Additionally, Hayiou-Thomas et al. [23] found in a large twin study that both environmental effects and genetics can impact early language development, and genetics appear to be the driving force behind individual differences in middle childhood and early adolescence.

Still, individual variability exists even in infants with family risk and much more needs to be learned to precisely predict future onset of DLD in an individual child, which may require taking into consideration both family and individual risk factors. Very few studies so far have examined early predictors of DLD in infancy, and most have focused on behavioral predictors. For example, Hsu & Iyer [24] found that lower usage of deictic gestures (pointing, reaching, showing, and giving) and conventional gestures (e.g., finger to lips for “hush”) as well as decreased vocabulary size at 15 months predicted risk for language impairment at 3 and 4.5 years of age. Similarly, Lüke et al. [25] observed that pointing ability at one year predicts language skills at 5 and 6 years of age, and this effect was mediated by comprehension of iconic gestures (e.g., opening and closing the extended index and middle finger to represent scissors) between 3–5 years. Other behavioral predictors are generally measured after 2 years of age and include receptive and expressive vocabulary, word combinations, and syntactic comprehension (see [14]). While these predictors can be useful, it is challenging to elicit and measure specific behaviors from infants. As an alternative method, neural predictors can provide richer and more nuanced data without requiring the active involvement of infants. Despite this advantage, neural predictors in infancy are yet to be examined.

Given recent findings that early neural speech processing in typically developing infants may predict later individual grammar skills [4], an important next step toward a sensitive and specific neural predictor of DLD is to examine, at the group level, neural speech processing in infants with vs. without first-degree family members who have a history of language delays and/or disorders (Low- vs High-Risk). This will provide a valuable window for us to uncover any already altered neural mechanisms that can later be tested as candidate predictors of atypical speech and language development using regression-based methods. Understanding the group-level differences between High-Risk and Low-Risk infants would not only allow for the expansion of language acquisition theories to encompass atypical development, but also help identify potentially much earlier markers for developmental speech and language disorders. This is a foundational step before we can further examine the exact predictive values of specific early markers for individual children’s later language outcomes.

### Language environment as a potential risk factor

In addition to family history, speech input from infants’ language environment may also have an important impact, as it has been repeatedly demonstrated to play a significant role in the language development of typically developing infants. In a seminal lab-based intervention study, only in-person language input in a social environment changed infants’ speech perception, demonstrating the importance of quality language input [26]. This effect was later replicated by analyzing daylong recordings made in typically developing infants’ home environments. The more infant-directed speech (IDS), characterized by more variable and increased vocal pitch, slower speech, and clearer articulation [27], in an infant’s home environment is observed, the stronger their later language outcome [28]. Further, by coaching parents to produce high-quality IDS during the *sensitive period* (6–12 months), researchers have demonstrated a significant intervention-related benefit in language development, when measured through both examining daylong audio recordings in infants’ home environments and parental surveys up to 30 months [29,30].

The effects of early speech quality and input have also been documented in clinical populations. For example, mothers exhibiting post-partum depression tend to speak less to their infants overall, and their infant-directed speech is reduced in both quantity and quality [31,32]. Mothers of infants at-risk for dyslexia have been found to hyperarticulate vowels in IDS significantly less than mothers of Low-Risk/typically developing infants [33]. It is thus possible that early speech input is significantly related to the family history risk factor, i.e., the quantity and/or quality of the speech input may be reduced if there is presence of speech and language disorder in the family. Such a relation would make the reduced speech input become a risk factor itself.

Therefore, it is important to evaluate the speech input in an infant’s language environment in parallel with the familial history risk factor. Language ENvironment Analysis (LENA) technology has provided an optimal method to assess infants’ speech input in a natural language environment, with the capacity for daylong recordings (i.e., up to 16 hours a day) using a small recorder that infants can wear. From these recordings, we will be able to calculate measures such as the total amount of speech infants hear and, more importantly, how much of that is infant-directed.

### Neural measures for assessing the *sensitive period* for phonetic learning

The recent advances in neural imaging technologies, particularly for infants [34], are now providing us the critical tools to start investigating and understanding the neural mechanisms important for speech learning during the *sensitive period*. Magnetoencephalography (MEG) is a safe and non-invasive method of evaluating functional brain activity with high temporal and spatial resolution [35]. Two neural indices are the focus of the current MEG measurement protocol, namely, the *mismatch response* (MMR) and the complex auditory brainstem response (cABR). First documented using the Electroencephalography (EEG), the *mismatch negativity/response (MMN/R)* is one of the most widely studied neural signatures that indexes neural sensitivity to sound change [36]. For infants during the *sensitive period*, converging evidence demonstrated similar MMN/R changes in both EEG and MEG [37–40]. MEG technology further allows a closer examination of underlying neural sources, or generators, of the *mismatch response* (MMR). So far, research has identified MMR in the left inferior frontal region (IF) to be a key process that holds higher predictive value for later language skills [3,4].

Another neural process, namely, the complex *auditory brainstem response (cABR)* has received increased research interest in recent years as a measure that characterizes the sensory encoding of the acoustic properties of complex sounds (e.g., speech sounds) in the early stages/lower level of auditory processing [41]. In adults, instead of a passive relay for information up through the auditory system, the cABR has been shown to be malleable by higher-level processes, such as early language and music experience, as well as short-term training [42–44]. Critically, our most recent study examined the frequency-following response (FFR), a component of cABR that tracks the periodic portion of the sound, in 7- and 11-month-old infants and found that both language and music experience affected the FFR [45]. This result suggests that cABR could also be an important neural process underlying the *sensitive period*. However, very limited data currently exists on infant cABR and there is a need to further understand changes in cABR with a focus on the *sensitive period* using a wide range of speech sounds. Further, methodologically, spatial information has become crucial for cABR, given recent research has suggested cortical contribution to FFR, originally thought to only come from subcortical regions [46]. Yet, most existing cABR relies on recording from a single EEG sensor, which cannot provide such spatial information in the cABR, making it crucial to gather MEG-recorded FFR/cABR.

### Current study and planned aims

The overarching goal of this current study is to build a large longitudinal dataset with infants at varying risk level for atypical language development, which will allow us to start addressing important research questions. Critically, this study gathers extensive information to characterize the familial risks not only by collecting detailed background information through parental surveys, but also by comprehensively assessing the language skills of the infants’ older sibling and examining the families’ home language environments. Infants’ neural processing of speech is recorded at three time points corresponding to the beginning (6 months), end (12 months) and a delayed end of the *sensitive period* (14 months) using magnetoencephalography (MEG) with MMR and cABR as target measures. Infant language development is further tracked with the MacArthur-Bates Communicative Development Inventory every three months between 18–30 months of age, followed by a comprehensive assessment of the same infants’ language skills when they reach elementary school age. In the Methods section, we document the study design, materials, and procedures used in data collection. The current study will be foundational in future potential clinical applications that would deploy brain-based measures very early in development to flag risk for later speech- and language-related disorders.

Our research team aims to address the series of questions listed below based on data collected in this study: Q1. Are there group-level differences between High- vs. Low-Risk infants already detectable through our target neural metrics around the *sensitive period* for phonetic learning? Q2. Are there differences in speech input, both quantity and quality, between High- vs. Low-Risk infants? Q3. Do High-Risk and Low-Risk infants present different language development trajectories? Q4. Can we improve prediction of individual infants’ speech and language outcomes at school entry by taking both family history and individual neural metrics into consideration?

## Materials and methods

### Participants

#### Eligibility.

Participants are infant-sibling pairs who share at least one biological parent and were enrolled by their parent(s). The following describes our enrollment criteria (Table 1).

**Table 1:** Enrollment criteria.

Inclusion Criteria	Exclusion Criteria
Infant is between 4.5 and 6 months of age at time of enrollment	Any of the infant’s immediate family members have major cognitive disorders, autism, or congenital hearing impairments
Infant has at least one biological sibling aged 3 or older (can be a half-sibling)	More than one language (English) is systematically used in the household
Infant was born within two weeks of their due date and their birth weight was between 6–10lbs	Infant and/or participating sibling has/have a known major health issue, frequent ear infections, or hearing loss

#### Recruitment.

Recruitment for this study began in April 2023 at the University of Washington (UW)’s Institute for Learning and Brain Sciences, after extensive piloting and training. Participating families were enrolled when their infants were between 4.5–5.5 months of age. Our main recruitment channels for enrollment included the UW Communication Studies Participant Pool, flyers, parent groups, and word of mouth. Additionally, to recruit participants at greater risk of atypical language development, advertisements were made to major SLP clinics and professional societies in the area. We aimed to have a balance of High- and Low-Risk infants included in the study.

This study involves multiple recruiting stages over the years for various measurements, and recruitment is ongoing. Initially, participating families were enrolled over the phone for four in-person visits spanning eight months. Recruitment for this stage spanned from April 6^th^, 2023 to July 3^rd^, 2025. During and at the end of this time period, the families are further recruited to participate in additional at-home measures (i.e., LENA and CDI). Recruitment for the first at-home measure (LENA recording) began on August 5^th^, 2023 and is expected to end on November 10^th^, 2025. Recruitment for the second additional measure (five CDIs, one every three months) began on April 25^th^, 2024 and is expected to end on August 17^th^, 2026. Finally, all participants consented to being contacted regarding future related research, and future contact will be made to recruit families back for the follow-up language assessment session when infants reach school entry age. Recruitment for this final measure will begin on October 16^th^, 2028, and is expected to end on February 5^th^, 2031.

#### Sample size and power analysis.

The proposed study aimed to include a minimum of 26 participants for each group (total minimum N = 52). An *a* *priori* power analysis was conducted based on a recent study that compared two groups of 6-month-old infants with different risk levels for Language and Learning Impairment in their auditory processing using EEG [47]. The main effect of group on the amplitude of an EEG feature was reported to have an effect size (Cohen’s d) of 0.706. Using the G*Power software [48], it shows that to detect a similar between-group effect at a level significance level of 0.05 with power above 0.80, the total sample size in the current study will need to be larger than 52 (i.e., 26 per group).

#### Participant characteristics.

We have enrolled 60 families in the study, and recruitment for multiple measures will continue to take place over the next few years (through 2031). 29 infants are female, and the average infant age at enrollment was 4.87 months (SD = 0.33, range = 4.24–5.59 mo.). 32 of the infants’ biological siblings are female, and their average age at enrollment was 4.64 years (SD = 1.81, range = 2.97–10.07 yr.). The sample was drawn from King County, which includes urban, suburban, and rural areas, and all testing was done at the Institute for Learning and Brain Sciences in Seattle, Washington. The majority of our sample is White (86.67%), and other participants are Asian, Black or African American, Middle Eastern, and/or Native American (6.67%, 6.67%, 1.67%, and 1.67%, respectively). 5% of participants are of Hispanic, Latinx, or Spanish origin. This sample reflects the diversity of the county population, with the exception of the Hispanic/Latinx and Asian groups [49]. This lack of diversity may have been caused by our requirement for participating families to be monolingual English-speakers. Furthermore, the median household income is $200,000 (M = $234,158, SD = $149,686, range = $10,000 - $750,000). This is higher than the county’s median family income of $154,463 [50], which could be due to families with higher-income jobs having greater flexibility to participate in longitudinal research studies. 100% of participants have at least one parent who completed high school, 93.33% have at least one parent with a degree from a college or technical school, and 36% have at least one parent with a graduate or professional degree. While children who are male and/or have lower socioeconomic status have been shown to be at a higher risk of DLD in population-based studies [5], in this study, we aimed to keep these characteristics equal across groups with family history as the only varying factor.

118 additional families completed a REDCap (Research Electronic Data Capture, [51,52]) screening form but were not enrolled. Reasons behind this included families not meeting study inclusion criteria (N = 31), not having risk factors during periods where these were prioritized in recruitment (N = 27), and not responding to calls from the research team (N = 24). Further details of the study’s screening process will be described in the following section. Five families who were enrolled in the study chose to unenroll, all before the first in-person measure (older sibling language assessment).

### Ethics statement

This study was approved by the University of Washington Institutional Review Board under STUDY00015376. Written consent was obtained from all participants for all study procedures.

### Overview

**Fig 1:**
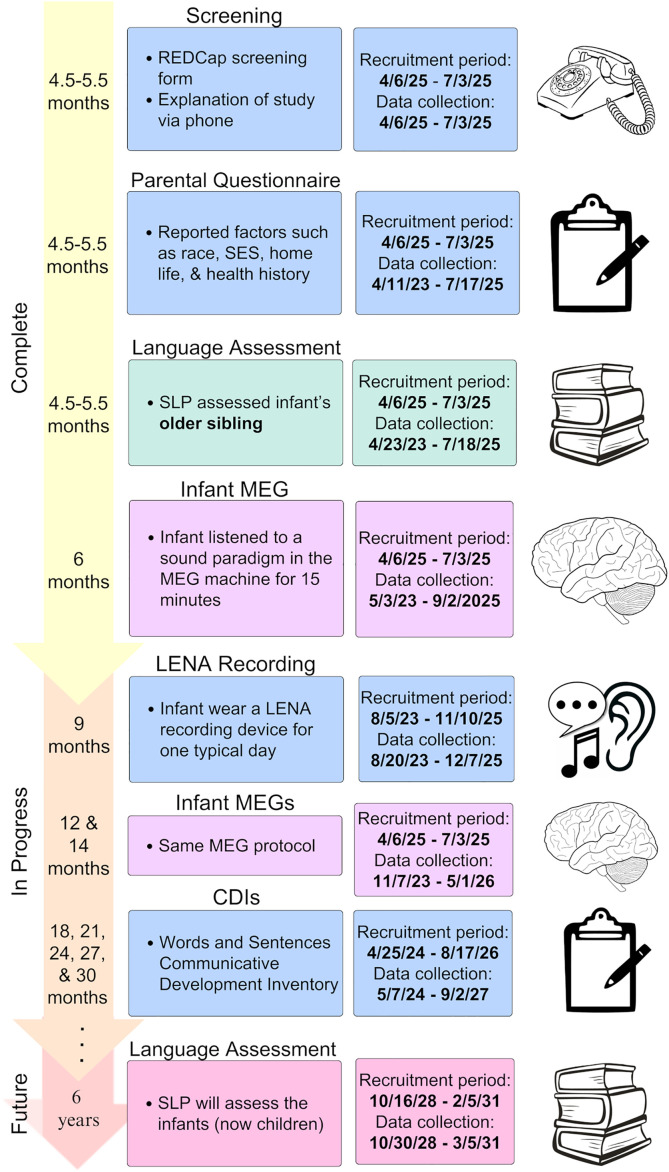
Study timeline.

Fig 1 illustrates the stages of this study. Interested parents were invited to complete a screening form and, if deemed eligible, were contacted via phone by a research assistant to discuss the study and enroll. Next, families were asked to complete a parental questionnaire before attending a brief Zoom call conducted by one of our licensed research SLPs, who verified their responses to the questionnaire and further discussed family health and language history with the parent(s). Then, the participating older sibling of the infant completed their language and behavioral assessment with the SLP during an in-lab visit before the infant turned 6 months of age. Next, the infants begin their magnetoencephalography (MEG) appointments, which are completed within two weeks of their six-, twelve-, and fourteen-month birthdays. At nine months, some families elect to be sent a Language ENvironment Analysis (LENA) recording device to record their infants’ home language environments for one typical day (around 12.5 hours and up to 16 hours). Additionally, families are asked to complete online versions of Communicative Development Inventories (CDIs) when their infants are 18, 21, 24, 27, and 30 months of age to track vocabulary and grammar development. Finally, when infants reach 6 years of age (school entry), they will be recruited to return for a language assessment. By incorporating extensive data collection methods in a longitudinal format, this study will provide a rich dataset that will allow researchers to thoroughly examine early predictors of (a)typical language development.

### Screening process

Interested families completed a REDCap screening form to provide information about eligibility criteria (ages, due dates, birth weights, etc.), risk status (language history), and contact information (email, phone number, and best times to contact). Parents could choose to skip any or all questions. Screening forms were reviewed by the research team, and qualifying families were called by a research assistant (RA). During the call, the RA went over each procedure in detail and emphasized the longitudinal nature of the study involving four in-person visits to the lab (UW I-LABS) and optional at-home measures. Furthermore, the RA went over the compensation system, which is done through e-gift cards. Participants could ask questions during this call and, if they decided to enroll, their first appointment (i.e., the older sibling’s language assessment) was scheduled. Upon enrollment, participants were sent a confirmation email containing detailed information and instructions about their upcoming appointment as well as links to their REDCap consent form and questionnaire. Screening forms from families who were not enrolled were deleted by an RA, who noted the reason for non-enrollment in a secure document.

### Informed consent

Participating families are provided detailed study information prior to and throughout their participation. First, before enrolling, a research assistant gave detailed descriptions of the in-person procedures of the study and answered any questions over the phone. Additionally, prior to each in-person appointment, the research SLP or RA goes over the appointment process again with the families. For the at-home components, detailed descriptions of the measure are provided through email. Participating families are reminded at each stage of the study that they can ask questions, end appointments early, and/or unenroll from the study at any time. Finally, a parent or legal guardian of each participant is required to complete a written consent form for all study procedures they elect to participate in detailing their involvement as well as the study’s purpose, risks, and benefits.

### Parental questionnaire

The parental questionnaire was administered through REDCap and collected information about the infants’ parents, such as their age, gender, race, education, economic status, living arrangements, and health history. It also included questions about the infants and their biological siblings, including their health and development (pregnancy/birth complications, milestones, medical screenings, medications, allergies, language development), childcare, auditory environment (additional languages, music exposure), and sleep schedules. Participants were asked to complete their questionnaire and consent form before meeting with the SLP to discuss their responses. Both structured and unstructured text data were generated from the questionnaire and securely stored on the ITHS REDCap server with access limited to team members who are actively involved in the project.

### Language and behavioral assessment: Older sibling

#### Pre-assessment zoom call.

During this Zoom call, the SLP verified the families’ answers to the parental questionnaire. This included confirming their children’s names and dates of birth, as well as their demographic, health, and language history. Risk factors for atypical language development, such as diagnoses and/or language struggles in childhood, were discussed and documented by the SLP. Finally, the SLP went over the procedure for the upcoming language assessment and answered any questions.

#### In-person assessment.

The assessments took place in a sound-attenuated booth, equipped with three SONY cameras and an Electro-Voice microphone. The video and audio streams were fed into a TV screen in an adjacent room before being recorded onto DVDs. Parents could observe the assessment in the adjacent room on the TV.

The materials used in the assessment session were: 1) The Goldman-Fristoe Test of Articulation, Third Edition (GFTA-3; [53]), which is a standardized, norm-referenced single-word speech sound assessment. It can be conducted on children ages 2–21 and takes 5–15 minutes to complete. 2) The Clinical Evaluation of Language Fundamentals, Fifth Edition (CELF-5; [54]), a standardized, norm-referenced test that assesses receptive and expressive aspects of language, including semantics, morphology, syntax, and pragmatics. It is designed for children ages 5–21 and takes 30–45 minutes to complete. Children ages 3–5 completed the Core Language Subtests of the Clinical Evaluation of Language Fundamentals Preschool-3. 3) The Test of Nonverbal Intelligence, Fourth Edition (TONI-4; [55]), which is an assessment designed to measure nonverbal intelligence in a format that can accommodate those with limited linguistic and/or motor skills. It can be administered to those ages six and older and takes 15–20 minutes to complete. This test was chosen over another measure of nonverbal intelligence, the Kaufman Brief Intelligence Test, due to its ability to accommodate 3-year-olds with an alternate version. Children 3–8 years of age completed the Primary Test of Nonverbal Intelligence (PTONI), which takes about 5–15 minutes.

Upon arriving, the SLP completed the Pragmatic Profile, a checklist that is part of the CELF, with input from the parent. Next, the child moved into the adjacent sound booth with the research SLP, and the assessment began with a pass-fail hearing screening using an MA 40 Air/Bone Conduction Audiometer (MAICO Diagnostics, Prairie, MN). Next, the research SLP administered the formal assessments in the following order: GFTA, CELF-5, and TONI-4 (or GFTA, CELP-P, and PTONI depending on the child’s age). Finally, the SLP elicited a language sample from the child for about 10–15 minutes. Different elicitation methods were used, including open-ended toys (e.g., baby doll for preschool age, Legos for school age), a wordless picture book (e.g., Goodnight, Gorilla for preschool age, Tuesday for school age), and an object brought by the child from their home (e.g., toy, stuffed animal, picture of a pet). Due to technical difficulties, three language samples were collected over Zoom, leading to poorer audio quality and less natural interaction. During testing, sticker sheets, breaks, and snacks were provided to meet each child’s individual needs and provide incentives.

Participants received compensation within a week. Additionally, parents were given the option to later receive a printed report of their child’s assessment results at their next lab visit. All recordings were de-identified and transferred to a secure server in digitized MP4 format for further data processing and analysis.

Physical DVD recordings and the physical copies of the test forms of the sessions are stored securely in a locked room that only the research team has access to. Digitized audio/video recordings of the sessions are stored in a local server that is password-protected. Tests scores were quality checked (see Data processing section) and entered into a secure REDCap database with limited access.

### Magnetoencephalography (MEG) sessions

For all MEG appointments, we follow the best practices outlined by Clarke et al. [56]. MEG assessments are scheduled early in the day following the infant’s nap and mealtime to increase the likelihood of a cheerful and cooperative infant during testing. Once the parent arrives and before beginning the session, the RA verbally checks in with the parent to ensure their infant does not need to be fed or changed, and inquires about any notable updates in their development since enrollment or the last visit. Next, the parent, infant, and research assistant simultaneously complete a “metal check”, in which they stand and move with the infant near the MEG machine sensors to ensure that no ferrous metal is present, as this metal can interfere with data quality. If interference is detected, its source is determined (clothing, jewelry, etc.) and parents are asked to remove the item from themselves or their infant if they are comfortable doing so before performing a second check. Adult- and infant-size scrubs are kept in the testing area, and parents are given the option to wear them if they do not have metal-free clothing on hand. Some parents have metal on their person that they are unable or unwilling to remove (e.g., jewelry or a permanent retainer); testing still occurs if it is small and the parent agrees to remain still in their seat.

After the metal check is complete, the first stage of the appointment begins: MEG preparation and digitization. During this segment, the infant is securely strapped into a highchair with Velcro straps while their parent sits out of sight. The parent is told they can interact with and soothe their infant whenever they like during the appointment, but are asked to remain just out of sight of the infant during the preparation process (i.e., sitting in a chair behind them).

To begin preparation, the MEG technician places a soft stretchy cap over the infant’s head with the strap sitting beneath their chin. Head Position Indicator (HPI) coils are taped to the cap in fixed locations to provide data about the infant’s head position relative to the MEG sensors. Next, the MEG technician uses a Polhemus 3-D magnetic digitizer pen to mark points on the infant’s head, including the locations of the HPI coils and the cardinal points. Electrocardiography (ECG) and electrooculography (EOG) electrodes are taped on the infant’s chest and back to measure muscle activity and heart rate, and on the outsides of their face to measure blinking activity. Next, small soft earbuds are placed in both of the infant’s ears and taped down with skin-safe tape to ensure they remain in place. Finally, a foam halo is placed around the infant’s head to secure the coils, protect their head, and reduce head movement in the machine. Throughout this stage, a research assistant speaks to the infant and engages them with various toys to keep them calm. The MEG technician takes detailed notes of the digitization process.

Once the preparation stage is complete, the infant is securely strapped into the MEG infant seat with Velcro straps, and the seat is adjusted so that the bottom of the dewar containing the superconducting sensors is near their shoulders. The parent is once again seated out of their infant’s sight but within reach of them. When the infant is secure, adjusted, and calm, one of six presentation sequences (randomly selected via RANDBETWEEN Excel function and tracked in a spreadsheet) initiates sound presentation, which employs a novel and time-efficient paradigm that presents speech sounds in 200 clusters, allowing for simultaneous assessment of cortical MMR and subcortical FFR. Each cluster has 8–14 tokens of the same speech stimuli. In each speech sound cluster, the presenting rate accelerates in the first few sounds and decelerates in the last few sounds, with jittered stimulus onset asynchrony to minimize predictability. See Cheng & Zhao [57] for further details.

During this time, a separate workstation records raw data from the MEG for later offline analysis. Throughout testing, a research assistant silently engages the infant with toys along with a video screen showing infant faces to keep them calm and alert. If the infant becomes fussy, the RA (and occasionally the parents) attempt to soothe them. If they do not calm down within a few minutes, the testing is paused or halted. If the infant remains calm, the appointment ends once the 15 minutes of trials finish. Video and audio are recorded from three different angles to provide context during data analysis. The audio/video recordings and the MEG recordings are uploaded to a password-protected server after each session. Additionally, the MEG technician monitors the infant’s behaviors and takes detailed notes. Participants are compensated for each completed MEG appointment, regardless of whether data collection was successful.

### Language ENvironment Analysis (LENA) recording at home

The LENA recorders are small recording devices that infants wear in specialized vests and are capable of recording up to 16 hours of audio in infants’ naturalistic sound environments. In addition, LENA software can process these recordings to compute a series of metrics to characterize the language input, including the number of words and conversational turns the infants experienced. LENA devices have many strengths. For example, they capture the full range of people who speak to and around infants (parents, grandparents, siblings, nannies, etc.). Additionally, they are a more subtle form of observation than lab visits, meaning participants are less likely to modify their natural behaviors due to bias. Finally, the day-long recording allows characterization of input across various contexts.

The recordings are scheduled for a typical weekend day within 3 weeks of infants’ nine-month birthdays, defined as days in which both caregivers are present for the majority of the day and the families are not sick, out of town, etc. Participating families are sent a package containing the LENA device, an infant vest specially made to hold the device, written instructions, and an activity diary. The instructions detail how to use the recorder and tell parents to remove the vest during naps, car rides, and baths for safety purposes, and to place the recorder near the infant in these situations if possible. To encourage participation in this measure, the option to have the recording package hand-delivered and retrieved by the research team is offered. Once the research team receives the returned LENA device, the family is compensated.

The LENA device is simple to use. After the families power on the recorders in the morning and place them in the infant vests, the devices automatically record for a full day and can turn themselves off if the storage limit is reached, though many parents manually end recordings after their infant has gone to bed. In addition to the recording, participants complete an activity diary to provide brief context for the recording in half-hour increments and make any relevant notes (e.g., breakfast, playing with sibling, nap time). Furthermore, families are provided a “Notice of Recording Script” which they can use to inform others that their child’s sound environment is being recorded for research. After their recording is complete, families mail the recorders, vests, and diaries back to the lab using our pre-paid return packaging. The encoded audio can only be uploaded using LENA’s secure cloud-based software onto our lab account. Data are automatically deleted from the devices after uploading and stored on our secure hard drive and server. Parents are assured that they may omit specific portions of the recording if desired.

### MacArthur-Bates Communicative Development Inventories

The Words and Sentences form of the MacArthur-Bates Communicative Development Inventories [58] is a validated and common way for evaluating language skills between ages 16–30 months [59], including the onset of certain vocabulary, word combinations, and grammatical forms. This survey method is advantageous as parents provide their assessment of their children based on continuous observation in diverse settings, as opposed to a brief laboratory visit that may not accurately represent a child’s behavior in their daily environment [60]. After families consent to participate in this measure prior to their infants’ 18-month birthday, REDCap is programmed to automatically send emails containing their unique survey links on their infants’ designated birthdays (18, 21, 24, 27, and 30 months of age). Parents can complete the survey remotely whenever it is convenient and may choose to save their partial response to finish later if needed. Additionally, if parents do not complete the form, a maximum of three reminder emails are automatically sent out every three days until the form is completed. If they still do not complete the form, this is considered a missing data point. Families are compensated for each completed survey, and their responses are stored in REDCap and scored using Python scripts. The use of CDIs to track our participants’ language development through 30 months of age will allow us to analyze how the various predictive factors we are examining correlate with their language scores.

### Comprehensive language assessment: Infant six-year follow-up

Parents will be recruited for an in-lab follow-up language assessment session when the infant participants have turned six years of age and completed kindergarten. This assessment will follow the same protocol as the older sibling language assessment with two modifications. Firstly, the SLP will have the parent(s) complete a brief follow-up survey similar to the parental questionnaire and provide informed consent in-person at the beginning of the appointment rather than via Zoom. Secondly, the Kaufman Brief Intelligence Test, Second Edition (KBIT-2; [61]) will be used in place of the TONI-4 for assessing non-verbal intelligence, which takes approximately 20 minutes to complete. This is because the TONI-4’s ability to accommodate 3-year-olds is no longer required and the KBIT will allow this dataset to merge with previous datasets using the same protocol. By following up with infant participants at six years of age to assess their language abilities, we can better examine how the measures taken in infancy predict language development and later language difficulties. The same data types and data storages will be used as those employed for behavioral assessment for older siblings.

### Piloting and training

Prior to data collection, pilot MEG appointments were completed with infants from each age group to determine the feasibility of the MEG appointment protocol. Additionally, both research assistants who ran the MEG appointments (first and second author) completed 15–20 practice sessions with infants in an MEG simulator to learn techniques and best practices in infant neuroimaging studies [56], ensuring the success of data collection.

### Data management plan

Data information associated with specific study procedures was described in each procedure section. All data files are de-identified and are designated a participant ID. The only link between identifiable information and de-identified data is securely stored in REDCap which only the research team has access to. The PI conducts weekly meetings with key study personnel to ensure the timeliness and quality of data entry.

Raw data are stored on a local password-protected server with highly controlled access as they may still contain private information, as allowed by the informed consent and the institutional certification. The informed consents contain language permitting secondary use with broad data sharing (de-identified) for general research use. Participating families will not be contacted or re-consented for future sharing or accessing data through repositories.

De-identified processed data, with parents’ consent for sharing, will be deposited at the Open Science Framework (OSF) repository under a project that can be findable and identifiable with a DOI created by OSF. The data deposit will occur when results generated from specific data are published. Data will be preserved within the repositories for at least three years following the completion of the research grant.

### Data preprocessing and planned analysis

#### Language assessment data preprocessing.

To ensure accuracy, results from the standardized assessment were calculated independently by a trained research assistant in addition to the research SLP. If a discrepancy was found, the independent rater and SLP discussed it until an agreement was reached, and the final scores were entered into REDCap. The language samples are transcribed using Codes for Human Analysis of Transcripts (CHAT; [62]) according to the manual and an adapted lab protocol that included relevant codes. For example, utterances are segmented based on the manual guidelines such as temporal cues (e.g., a pause of one second or more), terminal intonation cues, or grammatical structural cues. Additional rules include transcribing in all lowercase except for the pronoun “I” or proper nouns, using limited punctuation, and attaching preposed (e.g., “yes”, “well”) and postposed (e.g., “right?”) words to the main utterance.

After completing their transcription, the transcribers play the language sample video alongside it to check for any errors in areas such as segmentation, spelling, or speaker IDs. Revisions are made if necessary. At least 20% of all language samples will be transcribed by two RAs and reliability between transcribers will be calculated using the ‘RELY’ function in CLAN.

#### MEG preprocessing.

The raw MEG recordings are preprocessed for noise reduction using the MNE-python software v1.5 with default infant settings [56,63]. First, the oversampled temporal projection and temporal signal space separation are used to reduce sensor noise and external interference signals as well as to compensate for effects caused by the infants’ head movements during testing [64–66]. The MEG technicians mark which channels are bad, and only good channels are used to reconstruct sensor signals. Next, the signal-space projection method is used to isolate components of physiological artifacts, such as heartbeats [67]. The signal is then low-pass filtered at 50 Hz. Epochs are identified and selected from 100 ms before stimulus onset to 600 ms after stimulus onset to allow enough length to calculate MMR, with a baseline from −100–0 ms. Those that include a peak-to-peak amplitude larger than 4000 fT/cm for gradiometers and 4000 fT for magnetometers are excluded. For the Standards, 50 out of 100 total epochs are randomly selected to match the number of each Deviant. Finally, the evoked responses to each sound are derived by averaging across the accepted trials, respectively, and the MMR and FFR are then calculated.

#### Statistical analysis.

To address our listed four major planned research questions, two types of statistical approaches will be utilized: 1. Group-level comparisons between High-Risk vs. Low-Risk infants (Q1-3) and 2. Examining individual differences using regression-based statistical analysis (Q4). Within each approach, we will conduct both *a priori* hypothesis-based tests as well as data-driven tests. For example, to examine the differences in neural processing of speech between High-Risk vs. Low-Risk groups, we will first extract the two major neural indices (i.e., MMR and FFR) from *a priori* determined region of interests (ROIs) and time windows from the MEG recordings. Then, linear mixed-effect models will be used to assess the group-level differences by fitting group as a fixed effect in the model. Further, to explore additional differences outside of the target neural signatures, machine-learning based models will be employed to take the whole spatiotemporal patterns into consideration.

Currently, no data analysis has been performed and no results have been generated. Given the longitudinal nature and ongoing data collection of the study, we do not anticipate generating results until data collection from the CDIs are complete (September 2^nd^, 2027). Data collection is expected to end with the final follow-up language assessment (March 5^th^, 2031). Analysis and results of our planned questions will be documented in detail and communicated to all stakeholders in the subsequent publications.

## Discussion

Children with language difficulties are more likely to experience other socio-emotional and behavioral difficulties, even in adolescence and adulthood. These include anxiety, social phobia, ADHD, and depression [68–71], highlighting the need for early detection and intervention*.* The current study is the first known large-scale effort to longitudinally compare infants who are at Low vs. High risks of developmental language disorder (DLD) starting from the *sensitive period* for phonetic learning (6–12 months), with well-researched neural and behavior methods. This will produce a valuable longitudinal dataset that will allow researchers to better understand the early differences between these two groups and potentially the causes, predictors, and mechanisms of atypical language development. An improved understanding will better inform early detection and interventions for infants and children at-risk for communication disorders. This project is collecting data on numerous hypothesized predictors of language development, including factors describing infants’ family history/environment and factors describing infants’ own language development. Parents completed extensive questionnaires, and the infants’ siblings were assessed by a licensed research SLP. Additionally, infants are participating in three MEG scans at 6, 12, and 14 months, as well as a day-long home audio recording at nine months. Infants’ grammar and vocabulary development are being tracked from 18 to 30 months through MB-CDIs. Finally, language assessments will be completed when infants turn 6 years of age. As of July 3rd, 2025, 60 families have been enrolled in the study, and recruitment will continue through 2031. All participants have completed the parental questionnaire, the older sibling language assessment, and the infant 6-month MEG. The LENA recordings, 12- & 14-month MEGs, and CDIs are in progress and are expected to be completed in September of 2027. All families gave their consent to be contacted about the follow-up measure, which will begin in October of 2028. Beyond the planned research questions by the research team, this dataset will allow researchers to address a wide range of research questions to further advance the field, such as understanding the prevalence of late talking in High vs. Low-risk groups and its relationship with atypical language development.

Despite our effort in carefully designing the study and in widespread recruitment, the dataset has several limitations.

1. Attrition and missing data. While these are largely unavoidable due to the longitudinal nature of the data, great lengths are taken to minimize them. Firstly, flexible scheduling is provided for MEG appointments, including early mornings, weekends, and rescheduling options. Parents are asked to schedule appointments during mornings and after infants’ naps and mealtimes to improve infant cooperation. Additionally, an RA acts as a “toy waver” and distracted infants with toys to keep them calm throughout. Finally, parents are told they can end the appointment at any time to ensure their comfort. Despite these efforts, some infants (see Fig 1) are not able to cooperate for the length of the scan (e.g., not sitting still, fussing, and/or removing MEG cap). Furthermore, to reduce attrition in the home recording measure, information about the importance, usage, and security of the recording is included in the recruitment materials. In addition, the option to have the recording items hand-delivered and returned is offered. However, some families choose not to participate due to privacy concerns.
2. Large amounts of data require preprocessing that may involve labor-intensive manual coding. For example, transcription of language samples is a slow process that requires multiple transcribers to ensure reliability. Additionally, while LENA recorders can generate reports about infants’ language environments, their automated analysis has been shown not to correlate with human generated analysis [72], and manual analysis is time-consuming due to the length of the recordings. Future research can develop and validate novel automated methods.
3. Smaller and less diverse sample size. The extensive methods employed in this study allow for a deep investigation into each participating family but limit our sample size due to study scope. Additionally, requiring families to complete four in-person visits may have biased our sample toward a higher SES, as families who live in the city (Seattle) and have flexible work schedules are better able to participate. Because many of our participants are White and middle-class, future studies will likely need to deploy mobile recording methods to help reach a more diverse sample [73]. Future studies should also explore harmonizing existing datasets [3] to leverage state-of-the-art data science methods, such as machine-learning related methods.

In conclusion, this study represents an important first step in improving our understanding of language development in both Low- and High-Risk infants during the *sensitive period* for phonetic learning. This dataset will have a positive impact on treatments for children with language difficulties, and this protocol will allow other researchers to further strengthen our understanding of this topic through replication.
